# Trichlorido(5,5′-dimethyl-2,2′-bipyridine-κ^2^ *N*,*N*′)(methanol-κ*O*)indium(III)

**DOI:** 10.1107/S160053680803119X

**Published:** 2008-10-04

**Authors:** Khadijeh Kalateh, Roya Ahmadi, Amin Ebadi, Vahid Amani, Hamid Reza Khavasi

**Affiliations:** a Islamic Azad University, Shahr-e-Rey Branch, Tehran, Iran; b Department of Chemistry, Islamic Azad University, Kazeroon Branch, Kazeroon, Fars, Iran; c Department of Chemistry, Shahid Beheshti University, Tehran 1983963113, Iran

## Abstract

In the mol­ecule of the title compound, [InCl_3_(C_12_H_12_N_2_)(CH_4_O)], the In^III^ atom is six-coordinated in a distorted octa­hedral configuration by two N atoms from the chelating 5,5′-dimethyl-2,2′-bipyridine ligand, one O atom from a methanol molecule and three Cl atoms. In the crystal structure, inter­molecular O—H⋯Cl hydrogen bonds link the mol­ecules into chains parallel to [001].

## Related literature

For related literature, see: Ahmadi, Kalateh, Ebadi *et al.* (2008 ▶); Ahmadi, Khalighi *et al.* (2008 ▶); Amani *et al.* (2007 ▶); Khalighi *et al.* (2008 ▶); Khavasi *et al.* (2007 ▶, 2008 ▶); Tadayon Pour *et al.* (2008 ▶); Yousefi, Rashidi Vahid *et al.* (2008 ▶); Yousefi, Tadayon Pour *et al.* (2008 ▶). Yousefi, Khalighi *et al.* (2008 ▶). For related structures, see: Ilyukhin & Malyarick (1994 ▶); Malyarick *et al.* (1992 ▶); Nan *et al.* (1987 ▶); Ahmadi, Kalateh, Abedi *et al.* (2008 ▶).
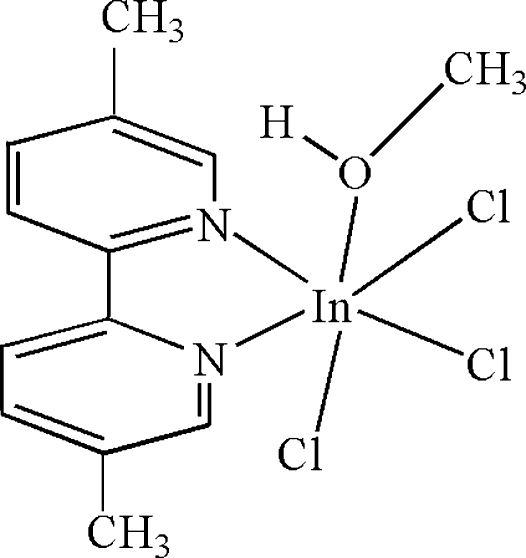


## Experimental

### 

#### Crystal data

[InCl_3_(C_12_H_12_N_2_)(CH_4_O)]
*M* *_r_* = 437.45
Monoclinic, 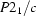

*a* = 10.9080 (6) Å
*b* = 11.2087 (7) Å
*c* = 13.3584 (8) Å
β = 107.211 (4)°
*V* = 1560.12 (16) Å^3^
*Z* = 4
Mo *K*α radiation
μ = 2.02 mm^−1^
*T* = 120 (2) K
0.17 × 0.15 × 0.10 mm

#### Data collection

Bruker SMART CCD area-detector diffractometer
Absorption correction: multi-scan (*SADABS*; Sheldrick, 1998 ▶) *T*_min_ = 0.729, *T*_max_ = 0.820
12144 measured reflections
4185 independent reflections
3716 reflections with *I* > 2σ(*I*)
*R*_int_ = 0.043

#### Refinement

*R*[*F* ^2^ > 2σ(*F* ^2^)] = 0.029
*wR*(*F* ^2^) = 0.062
*S* = 1.15
4185 reflections
185 parameters
H atoms treated by a mixture of independent and constrained refinement
Δρ_max_ = 0.92 e Å^−3^
Δρ_min_ = −0.68 e Å^−3^

### 

Data collection: *SMART* (Bruker, 1998 ▶); cell refinement: *SAINT* (Bruker, 1998 ▶); data reduction: *SAINT*; program(s) used to solve structure: *SHELXTL* (Sheldrick, 2008 ▶); program(s) used to refine structure: *SHELXTL*; molecular graphics: *ORTEP-3 for Windows* (Farrugia, 1997 ▶); software used to prepare material for publication: *WinGX* (Farrugia, 1999 ▶).

## Supplementary Material

Crystal structure: contains datablocks I, global. DOI: 10.1107/S160053680803119X/hk2541sup1.cif

Structure factors: contains datablocks I. DOI: 10.1107/S160053680803119X/hk2541Isup2.hkl

Additional supplementary materials:  crystallographic information; 3D view; checkCIF report

## Figures and Tables

**Table:** 

In1—Cl1	2.5015 (6)
In1—Cl2	2.4262 (6)
In1—Cl3	2.4080 (6)
In1—O1	2.2991 (19)
In1—N1	2.279 (2)
In1—N2	2.284 (2)

**Table:** 

Cl2—In1—Cl1	96.05 (2)
Cl3—In1—Cl1	100.89 (2)
Cl3—In1—Cl2	99.22 (2)
O1—In1—Cl1	169.20 (5)
O1—In1—Cl2	88.30 (5)
O1—In1—Cl3	88.11 (5)
N1—In1—Cl1	89.35 (5)
N1—In1—Cl2	93.30 (5)
N1—In1—Cl3	162.82 (6)
N1—In1—O1	80.51 (7)
N1—In1—N2	72.73 (7)
N2—In1—Cl1	87.68 (5)
N2—In1—Cl2	165.54 (5)
N2—In1—Cl3	93.76 (5)
N2—In1—O1	85.77 (7)

**Table 2:** Hydrogen-bond geometry (Å, °)

*D*—H⋯*A*	*D*—H	H⋯*A*	*D*⋯*A*	*D*—H⋯*A*
O1—H1*B*⋯Cl1^i^	0.83 (5)	2.29 (5)	3.115 (2)	174 (4)

Symmetry code: (i) 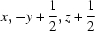.

**Fig. 1.**
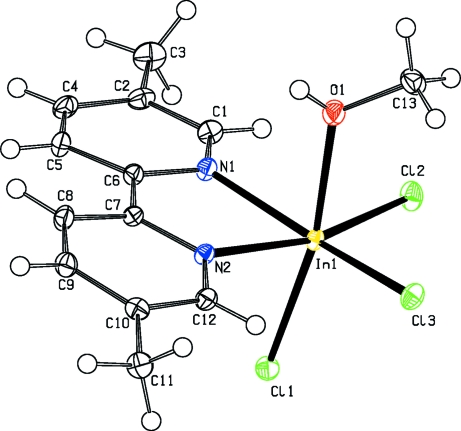
The molecular structure of the title molecule, with the atom-numbering scheme. Displacement ellipsoids are drawn at the 50% probability level.

**Fig. 2.**
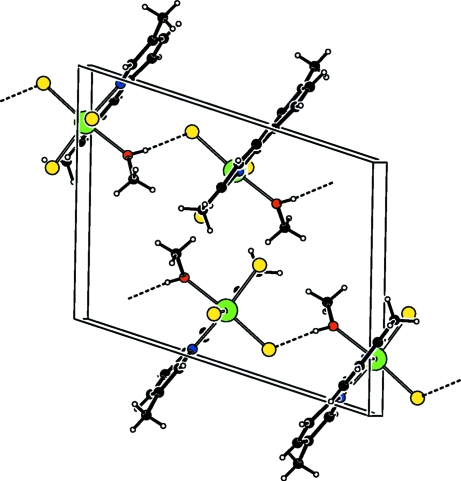
A partial packing diagram of the title compound. Hydrogen bonds are shown as dashed lines.

## supplementary crystallographic information

### Comment

Recently, we reported the syntheses and crystal structures of [Zn(5,5'-dmbpy)Cl_2_], (II), (Khalighi *et al.*, 2008), [Zn(6-mbpy)Cl_2_], (III), (Ahmadi, Kalateh *et al.*, 2008), [HgI_2_(4,4'-dmbpy)], (IV), (Yousefi, Tadayon Pour *et al.*, 2008), [Cd(5,5'-dmbpy)(µ-Cl)_2_]_n_, (V), (Ahmadi, Khalighi *et al.*, 2008), [Hg(5,5'-dmbpy)I_2_], (VI), (Tadayon Pour *et al.*, 2008), [Cu(5,5'-dcbpy)(en)(H_2_O)_2_].2.5H_2_O, (VII), (Yousefi, Khalighi *et al.*, 2008), [Hg(dmphen)I_2_], (VIII), (Yousefi, Rashidi Vahid *et al.*, 2008), and {[HgCl(dm4bt)]_2_(µ-Cl)_2_}, (IX), (Khavasi *et al.*, 2008). [where 5,5'-dmbpy is 5,5'-dimethyl-2,2'-bipyridine, 6-mbpy is 6-methyl-2,2'-bipyridine, 4,4'-dmbpy is 4,4'-dimethyl-2,2'-bi- pyridine, 5,5'-dcbpy is 2,2'-bipyridine-5,5'-dicarboxylate, en is ethylene- diamine, dmphen is 4,7-diphenyl-1,10-phenanthroline and dm4bt is 2,2'-dimethyl-4,4'-bithiazole]. We have also reported the syntheses and crystal structures of iron(III) complexes of [Fe(bipy)Cl_3_(DMSO)], (X) and [Fe(phen)Cl_3_(DMSO)], (XI), (Amani *et al.*, 2007) and [Fe(phen)Cl_3_(CH_3_OH)].CH_3_OH, (XII), (Khavasi *et al.*, 2007) [where bipy is 2,2'-bipyridine, DMSO is dimethyl sulfoxide and phen is 1,10-phenanthroline]. There are several In^III^ complexes, with formula, [In(N—N)Cl_3_(*L*)], (*L* = DMSO, H_2_O and EtOH), such as [In(bipy)Cl_3_(H_2_O)], (XIII), [In(bipy)Cl_3_(EtOH)], (XIV) and [In(bipy)Cl_3_(H_2_O)].H_2_O, (XV), (Malyarick *et al.*, 1992), [In(phen)Cl_3_(DMSO)], (XVI), (Nan *et al.*, 1987), [In(4,4'-dmbpy)Cl_3_(DMSO)],(XVII), (Ahmadi, Kalateh, Abedi *et al.*, 2008), [In(phen)Cl_3_(H_2_O)], (XVIII), and [In(phen)Cl_3_(EtOH)].EtOH, (XIX), (Ilyukhin & Malyarick, 1994) have been synthesized and characterized by single-crystal X-ray diffraction methods. We report herein the synthesis and crystal structure of the title compound, (I).

In the title compound, (Fig. 1), the In^III^ atom is six-coordinated in a distorted octahedral configuration by two N atoms from the chelating 5,5'-dimethyl-2,2'-bipyridine ligand, one O atom from one methanol and three Cl atoms. The In—Cl and In—N bond lengths and angles (Table 1) are within normal ranges, as in (XIII), (XIV), (XV) and (XVII).

In the crystal structure, intermolecular O—H···Cl hydrogen bonds (Table 2) link the molecules into chains (Fig. 2), in which they may be effective in the stabilization of the structure.

### Experimental

For the preparation of the title compound, (I), a solution of 5,5'-dimethyl -2,2'-bipyridine (0.20 g, 1.10 mmol) in methanol (20 ml) was added to a solution of InCl_3_.4H_2_O (0.16 g, 0.55 mmol) in methanol (50 ml) and the resulting colorless solution was stirred for 20 min at 313 K. This solution was left to evaporate slowly at room temperature. After one week, colorless block crystals of the title compound were isolated (yield; 0.18 g, 74.8%, m.p. <573 K).

### Refinement

H1B atom (for OH) was located in difference synthesis and refined isotropically [O-H = 0.83 (5) Å; U_iso_(H) = 0.036 (11) Å^2^]. The remaining H atoms were positioned geometrically, with C-H = 0.93 and 0.96 Å for aromatic and methyl H, respectively, and constrained to ride on their parent atoms with U_iso_(H) = 1.2U_eq_(C).

### Crystal data

**Table:**

[InCl_3_(C_12_H_12_N_2_)(CH_4_O)]	*F*(000) = 864
*M**_r_* = 437.45	*D*_x_ = 1.862 Mg m^−^^3^
Monoclinic, *P*2_1_/*c*	Mo *K*α radiation, λ = 0.71073 Å
Hall symbol: -P 2ybc	Cell parameters from 1324 reflections
*a* = 10.9080 (6) Å	θ = 2.0–29.2°
*b* = 11.2087 (7) Å	µ = 2.02 mm^−^^1^
*c* = 13.3584 (8) Å	*T* = 120 K
β = 107.211 (4)°	Block, colorless
*V* = 1560.12 (16) Å^3^	0.17 × 0.15 × 0.10 mm
*Z* = 4	

### Data collection

**Table:**

Bruker SMART CCD area-detector diffractometer	4185 independent reflections
Radiation source: fine-focus sealed tube	3716 reflections with *I* > 2σ(*I*)
graphite	*R*_int_ = 0.043
φ and ω scans	θ_max_ = 29.2°, θ_min_ = 2.0°
Absorption correction: multi-scan (SADABS; Sheldrick, 1998)	*h* = −14→14
*T*_min_ = 0.729, *T*_max_ = 0.820	*k* = −15→15
12144 measured reflections	*l* = −14→18

### Refinement

**Table:**

Refinement on *F*^2^	Primary atom site location: structure-invariant direct methods
Least-squares matrix: full	Secondary atom site location: difference Fourier map
*R*[*F*^2^ > 2σ(*F*^2^)] = 0.029	Hydrogen site location: inferred from neighbouring sites
*wR*(*F*^2^) = 0.062	H atoms treated by a mixture of independent and constrained refinement
*S* = 1.15	*w* = 1/[σ^2^(*F*_o_^2^) + (0.0209*P*)^2^ + 1.9934*P*] where *P* = (*F*_o_^2^ + 2*F*_c_^2^)/3
4185 reflections	(Δ/σ)_max_ = 0.017
185 parameters	Δρ_max_ = 0.92 e Å^−^^3^
0 restraints	Δρ_min_ = −0.68 e Å^−^^3^

### Special details

**Table:**

Geometry. All e.s.d.'s (except the e.s.d. in the dihedral angle between two l.s. planes) are estimated using the full covariance matrix. The cell e.s.d.'s are taken into account individually in the estimation of e.s.d.'s in distances, angles and torsion angles; correlations between e.s.d.'s in cell parameters are only used when they are defined by crystal symmetry. An approximate (isotropic) treatment of cell e.s.d.'s is used for estimating e.s.d.'s involving l.s. planes.
Refinement. Refinement of *F*^2^ against ALL reflections. The weighted *R*-factor *wR* and goodness of fit *S* are based on *F*^2^, conventional *R*-factors *R* are based on *F*, with *F* set to zero for negative *F*^2^. The threshold expression of *F*^2^ > σ(*F*^2^) is used only for calculating *R*-factors(gt) *etc*. and is not relevant to the choice of reflections for refinement. *R*-factors based on *F*^2^ are statistically about twice as large as those based on *F*, and *R*- factors based on ALL data will be even larger.

### Fractional atomic coordinates and isotropic or equivalent isotropic displacement parameters (Å^2^)

**Table:**

	*x*	*y*	*z*	*U*_iso_*/*U*_eq_	
In1	0.773908 (15)	0.320581 (14)	−0.004168 (13)	0.01003 (5)	
Cl1	0.87230 (6)	0.29569 (5)	−0.14993 (5)	0.01500 (11)	
Cl2	0.55271 (5)	0.31788 (6)	−0.11430 (5)	0.01766 (12)	
Cl3	0.79945 (6)	0.53204 (5)	0.02679 (5)	0.01588 (11)	
O1	0.69912 (19)	0.30834 (17)	0.13936 (16)	0.0178 (4)	
H1B	0.740 (4)	0.280 (4)	0.197 (4)	0.036 (11)*	
N1	0.7811 (2)	0.11891 (18)	0.01880 (17)	0.0126 (4)	
N2	0.97004 (19)	0.27552 (18)	0.10949 (16)	0.0109 (4)	
C1	0.6867 (2)	0.0450 (2)	−0.0335 (2)	0.0152 (5)	
H1	0.6098	0.0782	−0.0740	0.018*	
C2	0.6982 (3)	−0.0788 (2)	−0.0299 (2)	0.0160 (5)	
C3	0.5882 (3)	−0.1568 (2)	−0.0885 (2)	0.0222 (5)	
H3A	0.5655	−0.1386	−0.1619	0.027*	
H3B	0.5158	−0.1426	−0.0632	0.027*	
H3C	0.6132	−0.2391	−0.0777	0.027*	
C4	0.8152 (3)	−0.1254 (2)	0.0288 (2)	0.0172 (5)	
H4	0.8282	−0.2075	0.0310	0.021*	
C5	0.9129 (2)	−0.0502 (2)	0.0843 (2)	0.0161 (5)	
H5	0.9908	−0.0815	0.1247	0.019*	
C6	0.8929 (2)	0.0732 (2)	0.07869 (19)	0.0123 (4)	
C7	0.9932 (2)	0.1588 (2)	0.13432 (19)	0.0117 (4)	
C8	1.1071 (2)	0.1235 (2)	0.2067 (2)	0.0154 (5)	
H8	1.1202	0.0440	0.2269	0.018*	
C9	1.2017 (2)	0.2081 (2)	0.2486 (2)	0.0151 (5)	
H9	1.2782	0.1852	0.2974	0.018*	
C10	1.1821 (2)	0.3271 (2)	0.21787 (19)	0.0136 (4)	
C11	1.2857 (2)	0.4191 (2)	0.2546 (2)	0.0171 (5)	
H11A	1.2565	0.4812	0.2914	0.020*	
H11B	1.3060	0.4525	0.1952	0.020*	
H11C	1.3610	0.3824	0.3006	0.020*	
C12	1.0617 (2)	0.3568 (2)	0.15016 (19)	0.0137 (4)	
H12	1.0442	0.4365	0.1324	0.016*	
C13	0.6145 (3)	0.3950 (2)	0.1646 (2)	0.0186 (5)	
H13A	0.5359	0.3991	0.1083	0.022*	
H13B	0.6552	0.4718	0.1744	0.022*	
H13C	0.5963	0.3715	0.2278	0.022*	

### Atomic displacement parameters (Å^2^)

**Table:**

	*U*^11^	*U*^22^	*U*^33^	*U*^12^	*U*^13^	*U*^23^
In1	0.00974 (8)	0.00875 (8)	0.00997 (8)	0.00064 (6)	0.00040 (5)	0.00051 (6)
Cl1	0.0161 (3)	0.0161 (3)	0.0129 (3)	0.0011 (2)	0.0044 (2)	−0.0003 (2)
Cl2	0.0119 (2)	0.0182 (3)	0.0186 (3)	0.0012 (2)	−0.0019 (2)	0.0003 (2)
Cl3	0.0203 (3)	0.0100 (2)	0.0166 (3)	−0.0002 (2)	0.0043 (2)	−0.0008 (2)
O1	0.0205 (9)	0.0188 (9)	0.0152 (9)	0.0059 (7)	0.0071 (7)	0.0035 (7)
N1	0.0118 (9)	0.0110 (9)	0.0136 (10)	−0.0003 (7)	0.0015 (8)	0.0011 (7)
N2	0.0111 (9)	0.0103 (8)	0.0102 (9)	−0.0003 (7)	0.0017 (7)	0.0009 (7)
C1	0.0161 (11)	0.0140 (11)	0.0152 (12)	−0.0024 (9)	0.0038 (9)	−0.0015 (9)
C2	0.0211 (12)	0.0132 (11)	0.0154 (11)	−0.0040 (9)	0.0080 (10)	−0.0008 (9)
C3	0.0250 (13)	0.0181 (12)	0.0230 (14)	−0.0078 (10)	0.0062 (11)	−0.0056 (10)
C4	0.0246 (13)	0.0096 (10)	0.0204 (13)	−0.0005 (9)	0.0112 (10)	0.0004 (9)
C5	0.0177 (11)	0.0128 (10)	0.0181 (12)	0.0048 (9)	0.0059 (10)	0.0038 (9)
C6	0.0125 (11)	0.0128 (10)	0.0116 (11)	0.0017 (8)	0.0035 (9)	0.0016 (8)
C7	0.0124 (10)	0.0124 (10)	0.0106 (10)	0.0012 (8)	0.0037 (8)	0.0019 (8)
C8	0.0147 (11)	0.0136 (11)	0.0166 (12)	0.0030 (9)	0.0025 (9)	0.0033 (9)
C9	0.0119 (10)	0.0189 (11)	0.0127 (11)	0.0020 (9)	0.0006 (9)	0.0022 (9)
C10	0.0131 (10)	0.0164 (11)	0.0111 (10)	−0.0009 (9)	0.0033 (8)	−0.0015 (9)
C11	0.0124 (11)	0.0211 (12)	0.0149 (12)	−0.0022 (9)	−0.0004 (9)	0.0005 (10)
C12	0.0139 (11)	0.0150 (10)	0.0117 (11)	0.0010 (8)	0.0030 (9)	0.0023 (9)
C13	0.0183 (12)	0.0173 (11)	0.0227 (13)	0.0012 (9)	0.0098 (10)	−0.0030 (10)

### Geometric parameters (Å, °)

**Table:**

In1—Cl1	2.5015 (6)	C6—N1	1.347 (3)
In1—Cl2	2.4262 (6)	C6—C7	1.479 (3)
In1—Cl3	2.4080 (6)	C7—N2	1.355 (3)
In1—O1	2.2991 (19)	C7—C8	1.387 (3)
In1—N1	2.279 (2)	C8—C9	1.391 (4)
In1—N2	2.284 (2)	C8—H8	0.9300
O1—H1B	0.83 (5)	C9—C10	1.394 (4)
C1—N1	1.345 (3)	C9—H9	0.9300
C1—C2	1.393 (3)	C10—C12	1.397 (3)
C1—H1	0.9300	C10—C11	1.501 (3)
C2—C4	1.387 (4)	C11—H11A	0.9600
C2—C3	1.504 (4)	C11—H11B	0.9600
C3—H3A	0.9600	C11—H11C	0.9600
C3—H3B	0.9600	C12—N2	1.343 (3)
C3—H3C	0.9600	C12—H12	0.9300
C4—C5	1.389 (4)	C13—O1	1.447 (3)
C4—H4	0.9300	C13—H13A	0.9600
C5—C6	1.399 (3)	C13—H13B	0.9600
C5—H5	0.9300	C13—H13C	0.9600
			
Cl2—In1—Cl1	96.05 (2)	C2—C4—C5	120.4 (2)
Cl3—In1—Cl1	100.89 (2)	C2—C4—H4	119.8
Cl3—In1—Cl2	99.22 (2)	C5—C4—H4	119.8
O1—In1—Cl1	169.20 (5)	C4—C5—C6	119.2 (2)
O1—In1—Cl2	88.30 (5)	C4—C5—H5	120.4
O1—In1—Cl3	88.11 (5)	C6—C5—H5	120.4
N1—In1—Cl1	89.35 (5)	N1—C6—C5	120.5 (2)
N1—In1—Cl2	93.30 (5)	N1—C6—C7	117.2 (2)
N1—In1—Cl3	162.82 (6)	C5—C6—C7	122.3 (2)
N1—In1—O1	80.51 (7)	N2—C7—C8	120.6 (2)
N1—In1—N2	72.73 (7)	N2—C7—C6	116.6 (2)
N2—In1—Cl1	87.68 (5)	C8—C7—C6	122.8 (2)
N2—In1—Cl2	165.54 (5)	C7—C8—C9	119.4 (2)
N2—In1—Cl3	93.76 (5)	C7—C8—H8	120.3
N2—In1—O1	85.77 (7)	C9—C8—H8	120.3
In1—O1—H1B	125 (3)	C8—C9—C10	120.2 (2)
C13—O1—In1	124.22 (16)	C8—C9—H9	119.9
C13—O1—H1B	104 (3)	C10—C9—H9	119.9
C1—N1—In1	123.41 (17)	C9—C10—C12	116.8 (2)
C1—N1—C6	119.6 (2)	C9—C10—C11	121.8 (2)
C6—N1—In1	116.52 (16)	C12—C10—C11	121.4 (2)
C7—N2—In1	116.51 (16)	C10—C11—H11A	109.5
C12—N2—In1	123.91 (16)	C10—C11—H11B	109.5
C12—N2—C7	119.6 (2)	H11A—C11—H11B	109.5
N1—C1—C2	123.3 (3)	C10—C11—H11C	109.5
N1—C1—H1	118.4	H11A—C11—H11C	109.5
C2—C1—H1	118.4	H11B—C11—H11C	109.5
C4—C2—C1	116.9 (2)	N2—C12—C10	123.1 (2)
C4—C2—C3	122.3 (2)	N2—C12—H12	118.5
C1—C2—C3	120.8 (3)	C10—C12—H12	118.5
C2—C3—H3A	109.5	O1—C13—H13A	109.5
C2—C3—H3B	109.5	O1—C13—H13B	109.5
H3A—C3—H3B	109.5	H13A—C13—H13B	109.5
C2—C3—H3C	109.5	O1—C13—H13C	109.5
H3A—C3—H3C	109.5	H13A—C13—H13C	109.5
H3B—C3—H3C	109.5	H13B—C13—H13C	109.5
			
N1—In1—O1—C13	−152.9 (2)	N1—C1—C2—C3	178.8 (2)
N2—In1—O1—C13	133.9 (2)	C1—C2—C4—C5	2.5 (4)
Cl3—In1—O1—C13	40.01 (19)	C3—C2—C4—C5	−178.0 (2)
Cl2—In1—O1—C13	−59.27 (19)	C2—C4—C5—C6	−1.2 (4)
Cl1—In1—O1—C13	−173.3 (2)	C4—C5—C6—N1	−1.2 (4)
N2—In1—N1—C1	−175.9 (2)	C4—C5—C6—C7	−179.1 (2)
O1—In1—N1—C1	95.6 (2)	C5—C6—N1—C1	2.0 (4)
Cl3—In1—N1—C1	144.79 (17)	C7—C6—N1—C1	−180.0 (2)
Cl2—In1—N1—C1	7.9 (2)	C5—C6—N1—In1	−170.21 (19)
Cl1—In1—N1—C1	−88.11 (19)	C7—C6—N1—In1	7.8 (3)
N2—In1—N1—C6	−3.99 (17)	N1—C6—C7—N2	−8.3 (3)
O1—In1—N1—C6	−92.48 (18)	C5—C6—C7—N2	169.6 (2)
Cl3—In1—N1—C6	−43.3 (3)	N1—C6—C7—C8	173.2 (2)
Cl2—In1—N1—C6	179.80 (17)	C5—C6—C7—C8	−8.9 (4)
Cl1—In1—N1—C6	83.78 (17)	C8—C7—N2—C12	4.4 (4)
N1—In1—N2—C12	178.2 (2)	C6—C7—N2—C12	−174.2 (2)
O1—In1—N2—C12	−100.4 (2)	C8—C7—N2—In1	−176.82 (18)
Cl3—In1—N2—C12	−12.60 (19)	C6—C7—N2—In1	4.6 (3)
Cl2—In1—N2—C12	−166.46 (16)	N2—C7—C8—C9	−4.3 (4)
Cl1—In1—N2—C12	88.17 (19)	C6—C7—C8—C9	174.2 (2)
N1—In1—N2—C7	−0.55 (16)	C7—C8—C9—C10	−0.2 (4)
O1—In1—N2—C7	80.81 (17)	C8—C9—C10—C12	4.4 (4)
Cl3—In1—N2—C7	168.64 (17)	C8—C9—C10—C11	−174.9 (2)
Cl2—In1—N2—C7	14.8 (3)	C9—C10—C12—N2	−4.5 (4)
Cl1—In1—N2—C7	−90.59 (17)	C11—C10—C12—N2	174.9 (2)
C2—C1—N1—C6	−0.5 (4)	C10—C12—N2—C7	0.1 (4)
C2—C1—N1—In1	171.1 (2)	C10—C12—N2—In1	−178.61 (18)
N1—C1—C2—C4	−1.7 (4)		

### Hydrogen-bond geometry (Å, °)

**Table:**

*D*—H···*A*	*D*—H	H···*A*	*D*···*A*	*D*—H···*A*
O1—H1B···Cl1^i^	0.83 (5)	2.29 (5)	3.115 (2)	174 (4)

Symmetry codes: (i) *x*, −*y*+1/2, *z*+1/2.
